# Sunlight-activated composite TiO_2_-F-V-Mo materials for photodegradation of the organic pollutant methylene blue

**DOI:** 10.1016/j.heliyon.2024.e40489

**Published:** 2024-11-16

**Authors:** Ahmad Kassas, Batoul Dhaini, Israa Zahwa, Ramez Zayyat, Ali Shaito, Bassam Hussein, Mohamed Mouyane, Jérôme Bernard, David Houivet, Joumana Toufaily

**Affiliations:** a International University of Beirut, School of Engineering, Department of Industrial Engineering, Beirut, Lebanon; b Laboratory of Materials, Catalysis, Environment, and Analytical Methods (MCEMA), EDST, Lebanese University, Hadath, Beirut, Lebanon; c Laboratoire Universitaire des Sciences Appliquées de Cherbourg (LUSAC), EA 4253, Université de Caen -Normandie, BP 78, 50130, Cherbourg-en-Cotentin, France; d American University of Beirut, Department of Civil and Environmental Engineering, Lebanon; e Lebanese International University, School of Engineering, Department of Mechanical Engineering, Beirut, Lebanon; f Lebanese International University, School of Engineering, Department of Industrial Engineering, Beirut, Lebanon

**Keywords:** TiO_2_-LiF, Nanoparticles, Photocatalytic activity, Sunlight irradiation, Water treatment

## Abstract

F-doped TiO_2_ photocatalyst materials were prepared using solid-state and sol-gel synthesis with varying weight percentages of LiF or NH_4_F. Subsequently, molybdate and vanadium oxide were added to the prepared powders to create composite photocatalysts with reduced bandgap energy, enhancing light absorption in the visible region of the spectrum, which is essential for effective photocatalytic activity using sunlight. The synthesized powders were characterized by thermogravimetric analysis (TGA), scanning electron microscopy (SEM), and specific surface area using the BET method. The crystallization and anatase-to-rutile phase transformation of the powders were verified by X-ray diffraction (XRD), and the composite nanoparticles were further investigated by transmission electron microscopy (TEM). The photocatalytic activity of the F-doped commercial TiO_2_, sol-gel synthesized TiO_2_, and prepared composite powders was evaluated through the photocatalytic degradation of methylene blue (MB) in water under ultraviolet (UV), UV–visible, and sunlight irradiation. The results indicated that the incorporation of Molybdenum and Vanadium significantly influenced the photocatalytic efficiency by substantially reducing the bandgap of this composite photocatalyst. The LiF-doped TiO_2_ powders synthesized by sol-gel using TTIP, and doped with vanadium and molybdenum, activated by sunlight, exhibited high performance in MB degradation compared to commercial TiO_2_ and undoped synthesized TiO_2_ powders, achieving an optimal degradation rate of 7.26 × 10⁻⁹ mol per gram of photocatalyst.

## 1 Introduction

Environmental pollution is a growing concern worldwide, and efforts are being made to mitigate its impact. Pollution not only affects human health, but also has detrimental effects on plants, animals, and materials. Industrialization and urbanization are major contributors to pollution, causing severe harm to air, water, and soil quality. In addition, organic effluents from food industries, textiles, and paints exacerbate the already existing problems. It is essential to address pollution at its source and adopt sustainable practices to minimize its impact on the environment and living organisms [1].

Among the various forms of environmental pollution, water pollution is a significant concern. There are several methods available for the removal of contaminants from water. The diverse strategies employed for disinfection and pollutant elimination in water resources are categorized into physical, chemical, and biological methods. Physical techniques encompass processes such as filtration, sedimentation, distillation, and boiling, among others, aimed at wastewater purification. Chemical methods involve adsorption, flocculation, coagulation, chlorination, ozonation, and utilization of electromagnetic radiation like UV light, commonly utilized for this purpose. Biological processes, including slow sand filters and biologically active carbons, are also employed for decontamination. However, the rapid expansion of industry and modernization necessitate prioritizing more efficient water disinfection methods to address global purification challenges [2].

Photocatalysis, an advanced oxidation process, garners significant interest among researchers due to its advantageous features, including the potential for complete mineralization of pollutants, the use of sunlight irradiation, and its capability to function under mild operational conditions [3, 4]. Among the various photocatalysts, titanium dioxide (TiO₂) exhibits several advantageous properties, including exceptional chemical stability, abundant availability in the Earth's crust, high resistance to light-induced corrosion, significant thermal stability, and low toxicity [5, 6]. However, the wide electronic bandgap of TiO_2_ limits its absorption within the sunlight spectrum, resulting in less than 5 % utilization of sunlight energy [7]. Additionally, the low quantum efficiency of TiO_2_ in photochemistry stems from the pronounced recombination of electron-hole pairs [6, 8]. Therefore, enhancing the optical absorption and mitigating electron-hole recombination in TiO_2_ are crucial objectives for advancing its application in various fields.

Overcoming these limitations is achieved through non-metal doping, metal incorporation, and dye sensitization. These modifications enhance the absorption spectrum by reducing the bandgap and inhibit recombination reactions through efficient charge separation, thereby increasing catalytic activity [9]. Additionally, the performance of the photocatalysis is significantly influenced by particle size. In comparison to bulk TiO_2_, granular TiO_2_ nanoparticles possess an increased surface area and an expanded bandgap. These nanoparticles have a greater number of active sites, resulting in superior photocatalytic performance [10], [11].

Diverse techniques are available for the synthesis of TiO_2_ nanoparticles, with the sol-gel process emerging as particularly promising for the fabrication and preparation of both inorganic and organic-inorganic hybrid nanomaterials [12].

One of the commonly used dopants is lithium fluoride (LiF). Several studies have reported the enhanced photocatalytic activity of TiO_2_ doped with LiF, such as the work of Xia et al. (2017) who synthesized TiO_2_/LiF composite photocatalyst and demonstrated improved photocatalytic degradation of MB organic pollutants [13]. Zahwa et al. synthesized LiF-doped TiO_2_ powders using a flash combustion method and they demonstrated that the doped powders exhibited significantly enhanced performance in the degradation of methylene blue (MB) compared to both commercial TiO_2_ and undoped synthesized TiO_2_ powders [14].

Another approach for modifying TiO_2_ involves coupling with metal oxides; a method that has garnered significant attention for enhancing photocatalytic performance [15]. One possible configuration for enhancing the properties of TiO_2_ involves combining it with the semiconductor MoO_3_. This can lead to visible light absorption and increase available oxygen vacancies as well as the life span of the photoexcited electron–hole pairs [16,17]. In addition, extensive research has thoroughly examined the application of V_2_O_5_/TiO_2_ nanocomposite photocatalysts, owing to their remarkable photocatalytic capabilities [18]. The enhanced performance of V_2_O_5_/TiO_2_ photocatalysts is attributed to their narrow bandgap energy, expanded visible light absorption range, efficient separation of electron-hole pairs, and enhanced transfer of surface charge carriers [19].

Another recent development in photocatalysis is the activation of photocatalysts using alternate sunlight as a source of energy [20, 21]. This approach can reduce the dependence on expensive artificial light sources and enable the development of practical photocatalytic systems.

Nowadays, these semiconductor-based photocatalysts are activated by sunlight, UV, and UV–visible radiation. This study focuses on investigating the photocatalytic activity of a TiO_2_ catalyst doped with Fluorine as well as mixed oxides of TiO_2_ - MoO_3_ and/or V_2_O_5_, and the possibility of system activation by sunlight irradiation.

## 2 Materials and methods

All reagents used in this study were of analytical grade and were used as received. The reagents included Titanium (IV) oxide (anatase, 99 %, trace metal, Sigma Aldrich), Titanium tetraisopropoxide (TTiP) (98 % pure, Sigma Aldrich), Ammonium fluoride (NH_4_F, ACS reagent ≥98 %, Sigma Aldrich), Hydrofluoric acid (HF, 40 %), Oxalic acid (BDH), Citric acid, Ethanol (rectified 99 % Avonchem), Ammonium monovanadate (NH_4_VO_3_, Merck), Ammonium molybdate tetrahydrate [(NH_4_)Mo_7_O_27_.4H_2_O, FLUKA], and distilled water (Ultra-Pure).

The structure and microstructure of fluorinated TiO_2_ nanoparticles and the nanocomposite of TiO_2_-F doped with Vanadium and/or Molybdenum were investigated using various techniques, including X-Ray Diffraction (XRD), Transmission Electron Microscopy (TEM), Brunauer, Emmett, and Teller (BET) analysis, and Zeta potential measurement. To evaluate the photocatalytic activity of the synthesized and characterized photocatalysts, 0.1 g of prepared photocatalysts was added to Methylene Blue dye solution and subjected to UV light and UV–visible irradiation in a black chamber, as well as sunlight irradiation at room temperature under stirring.

## 3 Photocatalysts preparation

Photocatalysts nanocomposites are prepared via two different routes, solid-state, and sol-gel synthesis methods.

## 4 Solid-state synthesis

The Fluorinated Commercial TiO_2_ (T_C_) photocatalyst was prepared by stirring at room temperature T_C_ and Fluorine precursors (HF represented by F and NH_4_F indicated by F_N_) in distilled water for 24 h. T_C_F is then filtered, washed with distilled water several times, dried at 100 °C for 1h, and then calcined at 500 °C for 2 h. The Molybdate is incorporated into the T_C_F and T_C_F_N_ by mixing the substrate with 6 wt% of (NH_4_) Mo_7_O_27_.4H_2_O in acidic medium using citric acid. The sample was dried at 100 °C for 1 h and then calcined at 550 °C for 2 h. Other than Molybdate, Vanadium was incorporated into the structure of T_C_F and T_C_F_N_ by stirring with 2.5 wt% of NH_4_VO_3_ in a solution of oxalic acid. After filtration, the powders were dried at 100 °C for 1h and then calcined at 550 °C for 2 h.

### 4.1 Sol-gel synthesis

TTIP precursor and NH_4_F at 30 % of T_S_/F_N_ atomic ratio were dissolved in aqueous acetic acid solution and stirred for 24 h, dried at 67 °C for 1 h, and calcined at 500 °C for 2 h. T_S_F_N_ sample obtained was mixed with 6 wt% of (NH_4_) Mo_7_O_27_.4H_2_O and/or 2.5 wt% of NH_4_VO_3_ in an acidic medium by adding citric acid, filtered, and then dried at 100 °C for 1 h followed by calcination at 550 °C for 2 h.

## 5 Results and discussion

### 5.1 Structural and microstructure analysis

Investigation of crystalline structures by X-ray analyses were performed on three distinct sets of photocatalysts.

1. Commercial TiO_2_ fluorinated using HF (T_C_F), doped with Vanadium (T_C_FV), doped with Molybdenum (T_C_FMo), and doped with both Vanadium and Molybdenum (T_C_FVMo).
2. Commercial TiO_2_ fluorinated using NH_4_F (T_C_F_N_) and the doped variations T_C_F_N_V, T_C_F_N_Mo, and T_C_F_N_VMo.
3. Sol-gel synthesized photocatalyst TiO_2_ using TTIP and fluorinated using NH_4_F (T_S_F_N_), along with the doped alternatives T_S_F_N_V, T_S_F_N_Mo, and T_S_F_N_VMo.

**Fig. 1:**
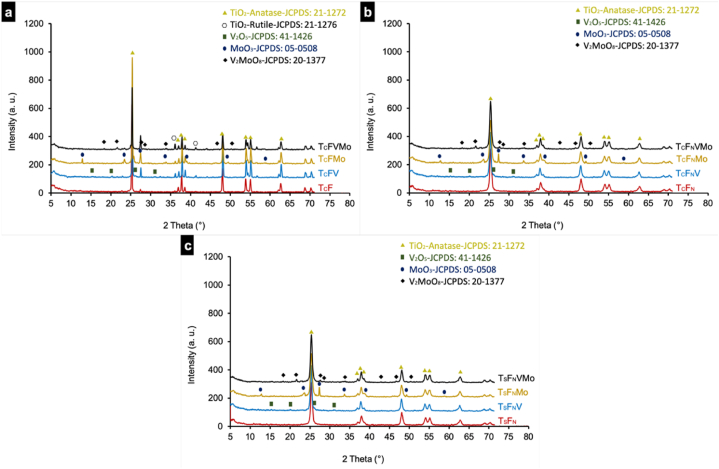
XRD patterns of the photocatalysts' powders prepared using different substrates (a) T_C_F (b) T_C_F_N_ (c) T_S_F_N_.

The X-ray diffractograms of the T_C_F (Fig. 1-a), T_C_F_N_ (Fig. 1-b) and T_S_F_N_ powder (Fig. 1-c), as well as the modified composites, are presented in Fig. 1. These diffractograms reveal the presence of TiO_2_ anatase solid phase and TiO_2_ rutile phase in certain samples, as indicated by the prominent peaks annotated in Fig. 1-a.

Additionally, the crystalline phase of V_2_O_5_ was detected in all diffractograms of the substrates prepared with Vanadium oxide. Furthermore, the appearance of peaks corresponding to the deposition of Molybdenum oxide (MoO_3_) on the crystalline phase of TiO_2_ particles was observed in the diffractograms of the T_C_FMo, T_C_F_N_Mo, and T_S_F_N_Mo samples.

Remarkably, when both Vanadium and Molybdenum were simultaneously added to prepare the composite photocatalyst (T_C_FVMo, T_C_F_N_VMo and T_S_F_N_VMo), a new phase, Vanadium Molybdenum oxide (V_2_MoO_8_), was detected. Regardless of the Ti and F sources used, samples containing Vanadium and/or Molybdenum exhibited the same phases as detected by XRD. The only distinguishing factor was the detection of the TiO_2_ rutile phase, which was observed in the diffractograms of T_C_ samples.

**Fig. 2:**
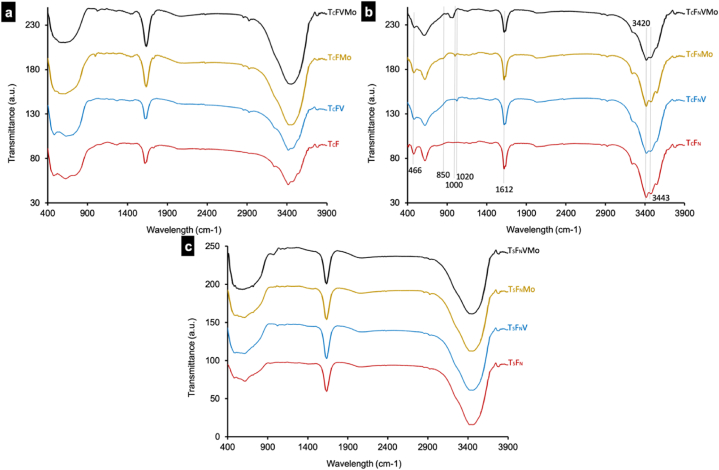
FT-IR analysis conducted on the photocatalysts' powders prepared using different substrates (a) T_C_F (b) T_C_F_N_ (c) T_S_F_N_.

The analysis of the FT-IR spectra (Fig. 2) obtained through the KBr method under ambient conditions, covering the wavenumber range of 400–3900 cm^−1^ for all the photocatalysts -T_C_F (Fig. 2-a), T_C_F_N_ (Fig. 2-b) and T_S_F_N_ (Fig. 2-c)- reveals the appearance of Ti-O bond at 466 cm^−1^ absorption peak. Meanwhile, the distinct peak at 1612 cm^−1^ is attributed to the deformation vibration of unbound water molecules, and the observed peak at 3420 cm^−1^ and 3443 cm^−1^ is associated with the stretching vibration of the functional groups -OH which favor the attachment of metal ions through electrostatic attraction [22]. The existence of these surface hydroxyl groups contributes significantly to the enhancement of photocatalytic activity by virtue of their interactions with photo-generated electron-hole pairs [23]. This interaction facilitates more efficient charge transfer and mitigates electron-hole recombination.

Regarding the peak at 445 cm^−1^, it can be attributed to the vibration arising from the Ti-F bonding [24]. However, this specific peak may not be easily visible due to potential interference from the broad peak within the wavenumber range of 400–900 cm^−1^. Furthermore, the vibration associated with the V=O bond manifests at 1020 cm^−1^. The absorption band around 850–1000 cm^−1^ corresponds to various Mo–O bonds [25].

**Fig. 3:**
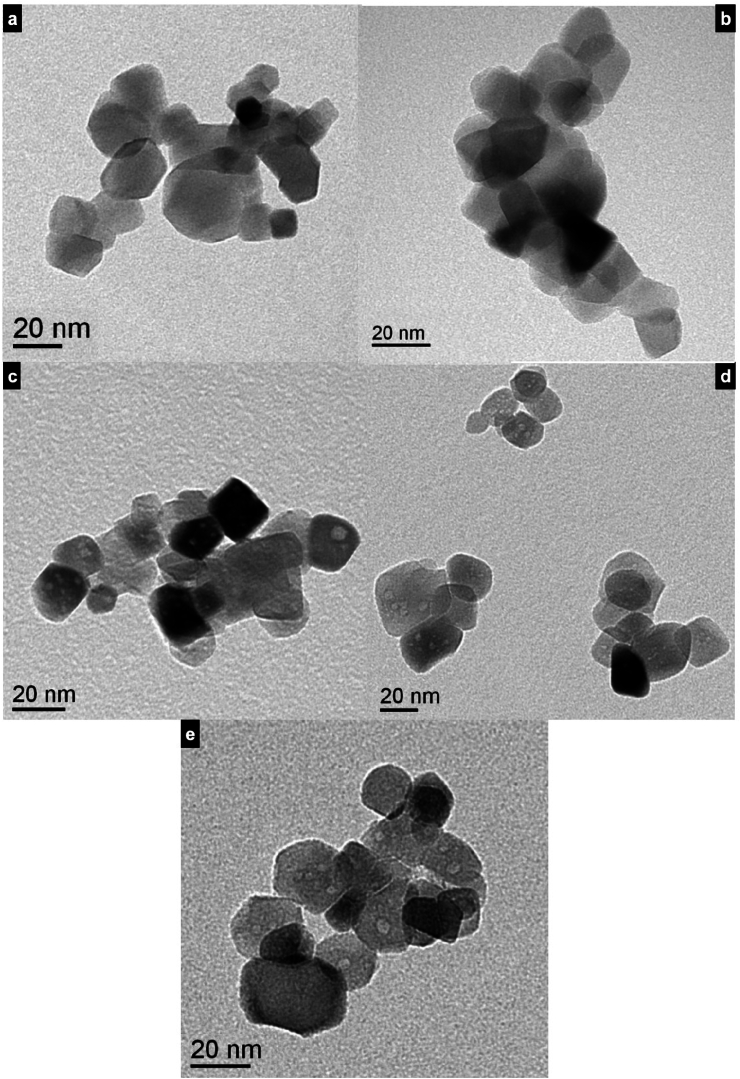
TEM Micrographs of the photocatalysts' powders: (a) T_C_F_N_, (b) T_S_F_N_, (c) T_S_F_N_Mo, (d) T_S_F_N_V, and (e) T_S_F_N_VMo.

Transmission Electron Microscope (TEM) analysis revealed that T_S_F_N_ doped with Vanadium and/or Molybdenum, exhibited full crystallinity. Micrographs of T_C_F_N_ photocatalyst (Fig. 3-a) displayed a nanocrystalline structure with non-uniform particle distribution and a size spectrum ranging from 13 nm to 36 nm.

In contrast, T_S_F_N_ powder (Fig. 3-b) demonstrated a narrow crystallized distribution of particle size, ranging from 15 to 18 nm. However, doping T_S_F_N_ with Mo (Fig. 3-c) resulted in a slight increase in particle size, with diameters ranging from 12 to 20 nm. Vanadium doping further increased particle size (Fig. 3-d), showing a more irregular shape distribution and a size range from 12 to 40 nm. The preparation of T_S_F_N_VMo composite photocatalyst led to a notable increase in particle size, ranging from 17 to 45 nm (Fig. 3-e). Remarkably, the particle morphology for T_S_F_N_ and T_S_F_N_Mo are cubic. However, the T_S_F_N_V shows a change in the morphology tending to be spherical. This irregular morphology of grains becomes more clear accompanied by a wider diameter size range and an increase in particles’ diameter.

To comprehend the observed variations in surface properties among T_C_F_N_, T_S_F_N_, T_S_F_N_V, T_S_F_N_Mo and T_S_F_N_VMo, it is crucial to investigate their surface charge and zeta potential. These parameters exert significant influence on the adsorption or interaction of both polar and non-polar substrates during photoreactions.

Analyzing and optimizing TF and its doped catalysts can establish correlations between surface charge, zeta potential, and catalytic performance. Higher values of zeta potential, whether positive or negative, indicate reduced particle agglomeration, enhancing the availability of particles for substrate adsorption and thus refining the interfacial properties of catalysts, thereby affecting their effectiveness [26].

**Fig. 4:**
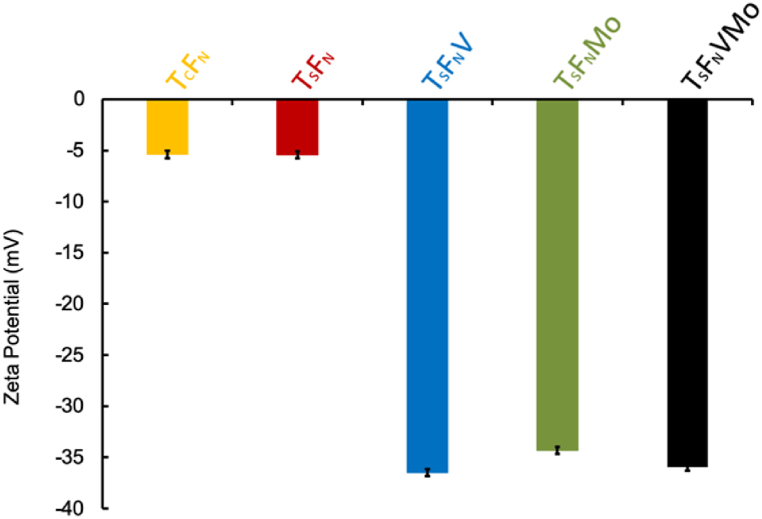
Zeta Potential of the commercial T_c_F_N_, sol-gel synthesized T_s_F_N_, T_s_F_N_V, T_s_F_N_Mo and T_s_F_N_VMo photocatalyst.

The measured surface charge of T_C_F_N_, T_S_F_N_, T_S_F_N_V, T_S_F_N_Mo and T_S_F_N_VMo particles in their aqueous dispersion were found to be −5.39, −5.43, −36.5, −34.3, and −35.9 mV, respectively (Fig. 4). There was no significant change in surface charge between T_C_F_N_ and sol-gel synthesized T_S_F_N_. However, after doping with V and Mo, their zeta potentials increased from −5.43 to −36.5 mV and to −34.3 mV, respectively. The variation in surface charge among these photocatalysts in the aqueous dispersion could be attributed to differences in surface adsorbed ionic species or surface acidity and basicity of the photocatalysts. The observed zeta potential values of the catalysts suggest that their aqueous suspensions are stable due to electrostatic repulsion between similarly charged species.

**Fig. 5:**
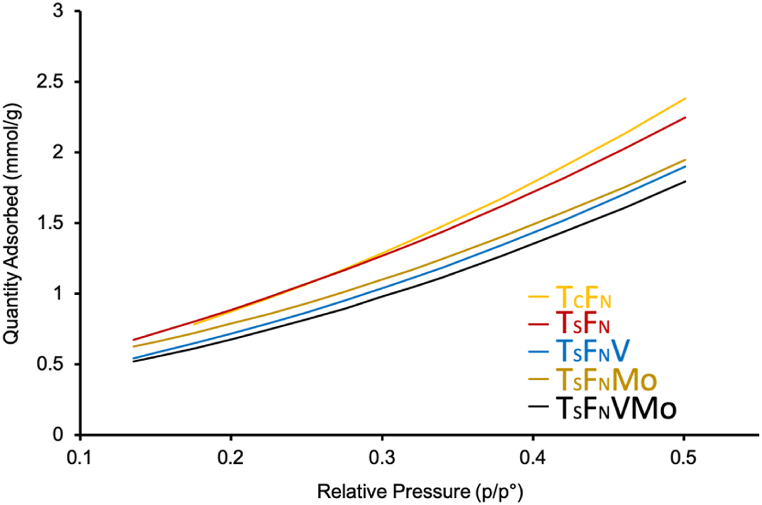
N_2_ adsorption using BET method at P/P_0_ between 0.15 and 0.5.

The nitrogen adsorption isotherm of the dried photocatalysts was analyzed by the BET method in a range of relative pressures P/P_0_ from 0.15 to 0.5 (Fig. 5). Type three isotherm, multilayer, and physical adsorbed-adsorbent interaction has been detected.

The adsorption decrease with the insertion of the dopant and the differences of the quantity of gas adsorbed became higher at elevated relative pressure. The lowest adsorbed gas quantity was detected for the T_S_F_N_VMo which can be correlated with the specific surface area analysis.

The BET surface area of as prepared T_S_F_N_ is found to be 134 m^2^/g which is less than the T_C_F_N_ counterpart, while the heteroatoms insertions of V, Mo and V + Mo has an impact on the photocatalyst specific surface area which decreases respectively from 134 m^2^/g to 115 m^2^/g, 81 m^2^/g, and 76 m^2^/g as indicated in Table 1 with a BET correlation coefficient of 0.99 obtained which indicates the experimental values are representative and acceptable.

**Table 1:** Specific surface area of the photocatalysts’ powders measured by BET analysis.

Photocatalyst	T_C_F_N_	T_S_F_N_	T_S_F_N_V	T_S_F_N_Mo	T_S_F_N_VMo
BET Surface (m^2^/g)	152.78 ± 1.79	134.02 ± 2.16	115.56 ± 2.64	80.96 ± 3.93	75.76 ± 4.39

The values of the specific surface area in Tale 1 are in accordance with the structural morphology and particle sizes observed in TEM images (Fig. 3). The decrease in S_BET_ of doped T_S_F_N_ with V, Mo and V + Mo accompanied by a particle size increase can be also explained by the partial coverage of T_S_F_N_ particles’ surface by V and Mo deposition.

### 5.2 Photocatalytic performance

**Fig. 6:**
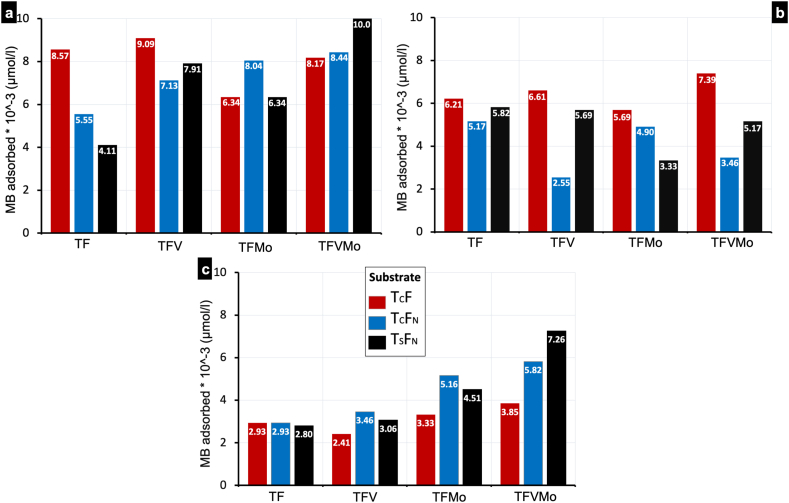
Comparison of MB adsorption by the prepared photocatalysts' powders under (a) UV–Visible, (b) UV, and (c) sunlight irradiation.

Three activation sources were used to evaluate the photocatalytic activity for adsorption of Methylene Blue (MB) by the prepared composite photocatalysts. The results are represented in Fig. 6.

The highest MB adsorption was recorded under UV–visible irradiation of the fluorinated substrate. The adsorption were 4.11, 5.55 and 8.57 × 10^−3^ μmol/g respectively for T_S_F_N_, T_C_F_N_ and T_C_F (Fig. 6-a), while the respective counterpart activated by Uv irradiation showed adsorption values of 5.82, 5.17, and 6.21 × 10^−3^ μmol/g (Fig. 6-b). The lowest adsorption was measured for the TF photocatalysts activated by sunlight as showed in Fig. 6-c (2.80, 2.93, and 2.93 × 10^−3^ μmol/g respectively).

Vanadium incorporation in the photocatalyst enhanced the MB adsorption under UV–Visible and the adsorption was detected for T_S_F_N_V (7.91 × 10^−3^ μmol/g) where the effect of the activation by visible and sunlight irradiation was very low and even inhibitive for MB adsorption which decreased relative to the counterpart activated by UV–visible light as in the case of T_C_F_N_V activated by Visible light (2.55 × 10^−3^ μmol/g) and T_C_FV activated by sunlight (2.41 × 10^−3^ μmol/g).

The histogram in Fig. 6 shows that the incorporation of molybdate onto the substrate has an effect dependent on the substrate used and the nature of the activation source. On the three substrates irradiated with UV, a negative effect on adsorption is observed, resulting in a decrease on the order of 0.5 × 10^−3^, 0.3 × 10^−3^, and 2.5 × 10^−3^ μmol/g, respectively, for the T_C_FMo, T_C_F_N_Mo, and T_S_F_N_Mo substrates.

UV–Visible activation decreased adsorption on the T_C_FMo photocatalyst compared to the undoped substrate T_C_F, while adsorption increased by approximately 2.5 × 10^−3^ and 2.2 × 10^−3^ μmol/g, respectively, for T_C_F_N_Mo and T_S_F_N_Mo. The importance of molybdenum incorporation was evident when the photocatalysts were activated by sunlight, where adsorption increased by approximately 0.4 × 10^−3^, 2.2 × 10^−3^, and 1.7 × 10^−3^ μmol/g, respectively, for T_C_FMo, T_C_F_N_Mo, and T_S_F_N_Mo. It is notable that the best adsorption effect is detected on the T_C_F_N_Mo photocatalyst activated by UV–Visible light, on the order of 8.04 × 10^−3^ μmol/g of photocatalyst.

The combination of molybdate and vanadium in the same photocatalyst improved adsorption on all substrates activated by sunlight (Fig. 6-c), with the maximum MB adsorption of 7.2 × 10^−3^ μmol/g, representing an increase of approximately 4.45 × 10^−3^ μmol/g. The second important observation is the notable activation by UV–Visible light of the same T_S_F_N_VMo photocatalyst, where adsorption is improved by approximately 5.9 × 10^−3^ μmol/g (Fig. 6-a).

The use of HF as a fluorine source showed high catalytic effect on the non-doped photocatalysts activated by Ultraviolet and UV–Visible irradiation, while the NH_4_F had positive impact when combined with Vanadium and Molybdate doped substrate as measured for T_S_F_N_VMo activated by Ultraviolet and sunlight irradiation. However, it had a negative impact when these photocatalysts are activated by UV–visible light.

Fig. 6-c shows that the most promising composite photocatalyst, for MB removal, activated by sunlight irradiation was the T_S_F_N_VMo with an adsorption capacity of 7.26 × 10^−3^ μmol/g.

### 5.3 Kinetic study and effect of temperature on photocatalyst functionality

**Fig. 7:**
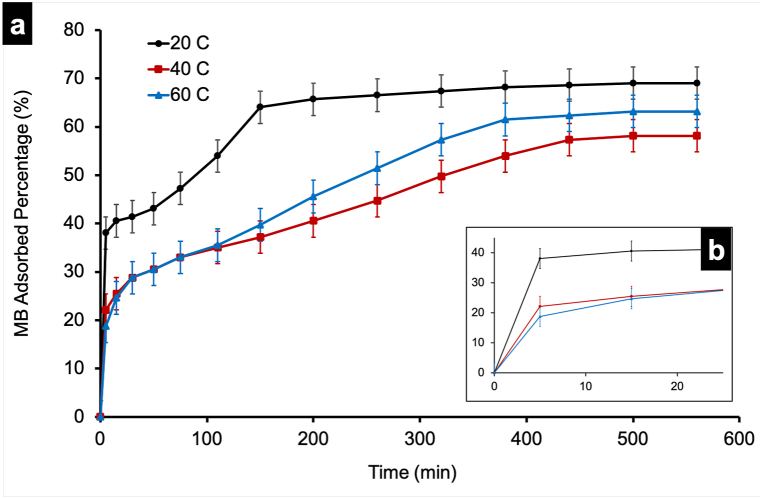
Temperature effect on the T_S_F_N_VMo samples (a) activated by UV–Visible irradiation and (b) a zoom between 0 and 25 min.

Observation of the results shown in Fig. 7-a indicates that the T_S_F_N_VMo photocatalyst exhibits a greater MB adsorption capacity at 20 °C compared to that at 40 °C and 60 °C, with an adsorption rate of about 7.06 % per minute detected initially reaching 38.04 % BM adsorption. After this value, the adsorption rate slows down, as indicated by the curve's trend, but then it accelerates again until it nears is close to saturation of adsorption sites, ultimately reaching a maximum of approximately 69 % after 500 min. This trend suggests that adsorption occurs in two stages: First, a rapid chemisorption of MB molecules forming a layer on the photocatalyst's active surface, followed by a slowdown, and then acceleration of MB adsorption, leading to the formation of a physisorbed layer above the first layer.

Increasing the medium temperature, adversely affects the adsorption capability of the photocatalyst, decreasing the adsorption rate from 7.06 %.min^−1^ to 4.4 %.min^−1^ as the temperature is raised from 20 °C to 40 °C, and further to 3.75 %.min^−1^ at 60 °C as indicated in Fig. 7-b. The initial rapid adsorption rate progressively slows down earlier as the temperature increases. This may be attributed to a reduction in the number of adsorption sites on the photocatalyst surface or increased bond vibrations induced by temperature elevation. The rate of physisorption decreases as the temperature rises from 20 °C to 40 and 60 °C, with saturation occurring more slowly. The maximum adsorption percentages are approximately 69 %, 63.19 %, and 58.16 % respectively at 20 °C, 60 °C, and 40 °C. Notably, the photocatalytic activity of the photocatalyst shows a minimum at 40 °C but increases again at 60 °C, though it remains lower than that at 20 °C. This composite photocatalyst demonstrates a temperature-dependent activity, leading to variations in degradation efficiency at different temperatures over time. Up to 25 min, the percentage of MB photodegradation at 40 °C was higher than that at 60 °C but the later shows higher percentage thereafter.

At low temperature, the photodegradation efficiency was found to be maximum. This is due the interaction of the dyes’ molecule with the photocatalyst surface. Hence, the adsorption increases which favors the photodegradation of dyes [27].

### 5.4 Measurements of UV–vis spectroscopy, in diffuse reflectance mode (DRS)

**Fig. 8:**
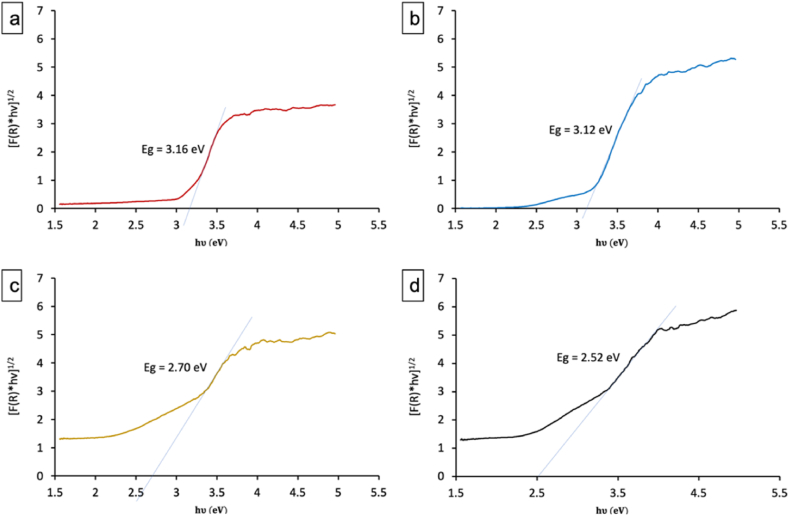
Tauc Plot Analysis and bandgap measurements for: a) T_C_F_N_, b)T_S_F_N_, c) T_C_FVMo and d) T_S_F_N_VMo samples.

The Kubelka-Munk theory and Tauc plot analysis were employed to determine the bandgap energy (Eg) of the samples from their optical properties. The Eg values were derived by extrapolating the linear region of the Tauc plot to the intersection with the photon energy (hν) axis. A slight variation in the TiO_2_ source had minimal influence on the bandgap, with T_C_F_N_ (Fig. 8-a) and T_S_F_N_ (Fig. 8-b) showing similar values of 3.16 ± 0.02 eV and 3.12 ± 0.02 eV, respectively. However, doping with vanadium and molybdate led to a significant reduction in the bandgap. In the case of T_S_F_N_ and T_S_F_N_VMo (Fig. 8-d), Eg decreased from 3.12 ± 0.02 eV to 2.52 ± 0.02 eV. Moreover, the [F(R) ∗ hν]^1/2^ as function of hν plot indicated the presence of two distinct bandgaps, which may be attributed to the coexistence of a VMoO_5_ phase alongside fluorine-doped TiO_2_. Similarly, doping with vanadium and molybdate in the T_C_FVMo sample reduced the bandgap, resulting in an Eg of 2.70 ± 0.02 eV (Fig. 8-c).

**Fig. 9:**
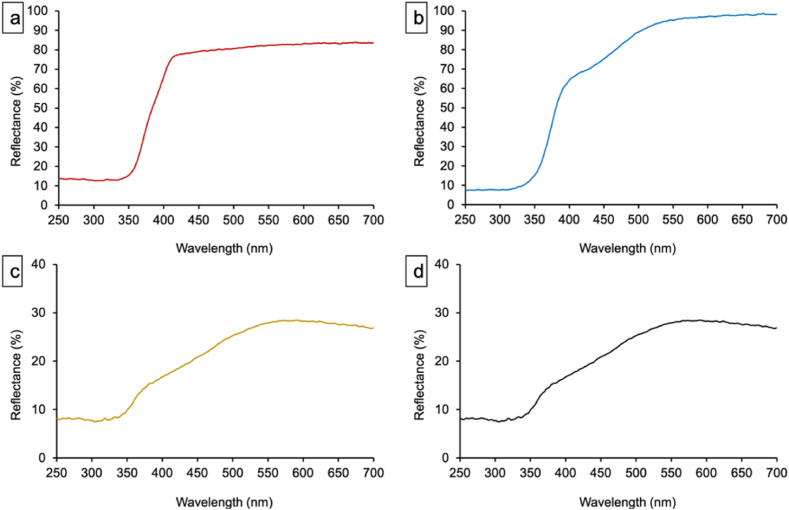
Diffuse Reflectance spectra for: a) T_C_F_N_, b)T_S_F_N_, c) T_C_FVMo and d) T_S_F_N_VMo samples.

UV–Vis diffuse reflectance spectroscopy (DRS) was employed to investigate the diffuse reflectance spectra of T_C_F_N_, T_S_F_N_, T_C_FVMo, and T_S_F_N_VMo powders (Fig. 9). The fluorine-doped TiO_2_ (T_C_F_N_) sample exhibited an increase in reflectance from 12.5 % to 78 % across the wavelength range of 335–420 nm (Fig. 9-a). In contrast, the T_S_F_N_ powder showed two distinct reflectance regions: from 313 to 420 nm, the reflectance increased from 7.5 % to approximately 68 %, and from 420 to 600 nm, the reflectance reached up to 98 % (Fig. 9-b).

Upon doping with vanadium and molybdate, the T_C_FVMo and T_S_F_N_VMo samples displayed similar reflectance spectra, with reflectance values ranging from 7.7 % to 28 % over the 313–575 nm wavelength range (Fig. 9-c and d). The reduced reflectance in these doped samples is attributed to enhanced light absorption and scattering effects caused by the incorporation of vanadium and molybdate, leading to decreased reflectance.

**Fig. 10:**
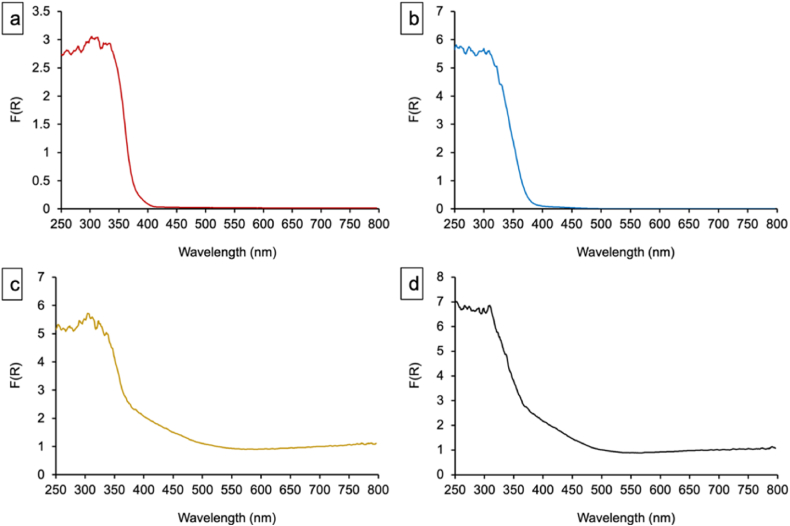
Kubelka-Munk function for the following samples: a) T_C_F_N_, b)T_S_F_N_, c) T_C_FVMo and d) T_S_F_N_VMo.

The Kubelka-Munk function, defined by the equation: $F\left(R\right)=\frac{{\left(1-R\right)}^{2}}{2R}$, is approximately proportional to the absorption coefficient and thus provides valuable information about the absorption properties of a sample. Fig. 10 illustrates the Kubelka-Munk function for T_C_F_N_, T_S_F_N_, T_C_FVMo, and T_S_F_N_VMo samples. The insets highlight the function within the visible range of the electromagnetic spectrum, spanning from 330 to 415 nm for T_C_F_N_ (Fig. 10-a) and from 313 to 415 nm for T_S_F_N_ (Fig. 10-b). In contrast, the vanadium- and molybdenum-doped catalysts, T_C_FVMo (Fig. 10-c) and T_S_F_N_VMo (Fig. 10-d), exhibit a broader absorbance range, extending from 313 to 570 nm. The increased absorption, particularly in the T_S_F_N_VMo sample compared to T_S_F_N_, reflects the impact of vanadium and molybdenum doping.

### 5.5 Absorbance as function of photocatalytic treatment time of MB solution treated with T_S_F_N_VMo

A 100 mL sample of 5 ppm methylene blue (MB) solution was mixed with 0.2 g of T_S_F_N_VMo catalyst and irradiated under UV–Visible light for 10 and 40 min. Post-treatment, the samples were subjected to centrifugation to separate the catalyst from the solution, and the resulting supernatant was analyzed using a UV–Visible spectrophotometer (Agilent® 8453).

**Fig. 11:**
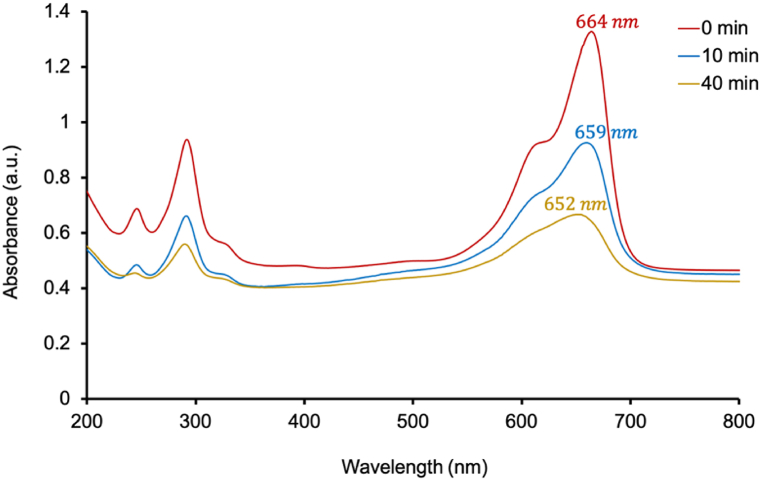
Time-dependent absorbance of a 100 mL, 5 ppm methylene blue (MB) solution treated with 0.1 g of T_S_F_N_VMO.

The absorbance spectra indicated a marked decrease over time, with a reduction from 1.327 at 0 min to 0.665 after 40 min of irradiation (Fig. 11). This reduction exceeds the changes observed in a control experiment conducted under identical conditions without light exposure (in the dark), where the absorbance only decreased slightly from 1.327 to 1.314 after 40 min.

Additionally, the wavelength corresponding to maximum absorbance shifted from 664 nm at 0 min to 659 nm at 10 min, and further to 652 nm after 40 min, suggesting significant photodegradation of MB molecules during the treatment.

## 6 Conclusions

The TiO_2_-F composite photocatalyst, doped with MoO_3_ and/or V_2_O_5_, was synthesized using both solid-solid and sol-gel methods. Structural analysis confirmed the successful incorporation of Molybdate and Vanadium into the structure, resulting in the formation of a new phase, Vanadium Molybdenum oxide (V_2_MoO_8_), in the TFVMo photocatalysts. FTIR analysis further substantiated the presence of these elements, indicating characteristic peaks corresponding to the vibration types of Ti-F, V=O, and Mo–O bonds, as well as the stretching vibration of -OH functional groups.

The aqueous dispersion of T_S_F_N_VMo particles exhibited a high surface charge, measured at −35.9 mV, indicating stability and good adsorption properties of the catalysts in suspension. BET analysis on dried photocatalysts revealed a type three isotherm, indicating multilayer, physically adsorbed-adsorbent interaction. However, the insertion of Vanadium and Molybdenum had a negative impact on the specific surface area of the photocatalyst, resulting in a decrease of 76 m^2^/g for the T_S_F_N_VMo composite.

Notably, the combination of molybdate and vanadium in the same photocatalyst improved adsorption on all substrates. The highest activity was achieved with UV–Visible light activation of the T_S_F_N_VMo photocatalyst, exhibiting an adsorption of 10 × 10^−3^ μmol/g. Conversely, when irradiated by sunlight, the same composite photocatalysts showed relatively maximum MB adsorption of 7.2 × 10^−3^ μmol/g. Furthermore, the photocatalyst demonstrated temperature-dependent activity, with variations in degradation efficiency observed at different temperatures over time, indicating maximum photodegradation efficiency at low temperatures.

In conclusion, this research underscores the significant role of vanadium and molybdenum doping in enhancing the optical properties and photocatalytic efficiency of fluorine-doped TiO₂. The reduction of the bandgap energy from 3.12 eV to 2.52 eV in the T_S_F_N_VMo catalyst not only broadens its light absorption spectrum into the visible region but also enables the activation of photocatalytic activity under sunlight. This characteristic is crucial for practical applications in environmental remediation, as it allows for efficient degradation of pollutants like methylene blue using readily available solar energy, making these doped catalysts highly valuable for sustainable photocatalytic processes.

## CRediT authorship contribution statement

**Ahmad Kassas:** Writing – review & editing, Validation, Supervision, Investigation, Formal analysis. **Batoul Dhaini:** Investigation, Formal analysis. **Israa Zahwa:** Investigation, Formal analysis. **Ramez Zayyat:** Resources, Formal analysis. **Ali Shaito:** Writing – review & editing, Investigation. **Bassam Hussein:** Validation, Resources, Project administration, Methodology. **Mohamed Mouyane:** Validation, Investigation, Formal analysis. **Jérôme Bernard:** Validation, Methodology, Investigation. **David Houivet:** Supervision, Investigation, Formal analysis. **Joumana Toufaily:** Validation, Supervision, Funding acquisition, Data curation, Conceptualization.

## Data and code availability statement

Data will be made available on request.

## Declaration of competing interest

The authors declare that they have no known competing financial interests or personal relationships that could have appeared to influence the work reported in this paper.

## References

[1] David noel S., Rajan M. (2014). Impact of dyeing industry effluent on groundwater quality by water quality index and correlation analysis. Journal of Pollution Effects & Control.

[2] Singh P., Shandilya P., Raizada P., Sudhaik A., Rahmani-Sani A., Hosseini-Bandegharaei A. (2020). Review on various strategies for enhancing photocatalytic activity of graphene based nanocomposites for water purification. Arab. J. Chem..

[3] Lu Y., Zhang H., Fan D., Chen Z., Yang X. (2022). Coupling solar-driven photothermal effect into photocatalysis for sustainable water treatment. J. Hazard Mater..

[4] Saianand G., Gopalan A.I., Wang L., Venkatramanan K., Roy V.A., Sonar P., Naidu R. (2022). Conducting polymer based visible light photocatalytic composites for pollutant removal: progress and prospects. Environ. Technol. Innovat..

[5] Chen X., Mao S.S. (2007). Titanium dioxide nanomaterials: synthesis, properties, modifications, and applications. Chem. Rev..

[6] Ullattil S.G., Narendranath S.B., Pillai S.C., Periyat P. (2018). Black TiO_2_ nanomaterials: a review of recent advances. Chem. Eng. J..

[7] Chen X., Liu L., Huang F. (2015). Black titanium dioxide (TiO_2_) nanomaterials. Chem. Soc. Rev..

[8] Thompson T.L., Yates J.T. (2006). Surface science studies of the photoactivation of TiO_2_ new photochemical processes. Chem. Rev..

[9] Peiris S., de Silva H.B., Ranasinghe K.N., Bandara S.V., Perera I.R. (2021). Recent development and future prospects of TiO_2_ photocatalysis. J. Chin. Chem. Soc..

[10] Tian J., Zhao Z., Kumar A., Boughton R.I., Liu H. (2014). Recent progress in design, synthesis, and applications of one-dimensional TiO_2_ nanostructured surface heterostructures: a review. Chem. Soc. Rev..

[11] Bianchi C.L., Gatto S., Pirola C., Naldoni A., Di Michele A., Cerrato G., Capucci V. (2014). Photocatalytic degradation of acetone, acetaldehyde and toluene in gas-phase: comparison between nano and micro-sized TiO_2_. Appl. Catal. B Environ..

[12] Esposito S. (2019). “Traditional” sol-gel chemistry as a powerful tool for the preparation of supported metal and metal oxide catalysts. Materials.

[13] Xia X., Liu H., Yu D., Bao Y., Gao Y. (2017). Remarkable absorbability enhancement of TiO_2_ through surface modification by LiF. Sci. Adv. Mater..

[14] Zahwa I., Mouyane M., Kassas A., Kamlo A.N., Moslah C., Navas J., Houivet D. (2024). Flash combustion synthesis using two different fuels and characterization of LiF-doped TiO_2_ for the photocatalytic applications. Open Ceramics.

[15] Bai S., Liu H., Sun J., Tian Y., Chen S., Song J., Liu C.C. (2015). Improvement of TiO_2_ photocatalytic properties under visible light by WO_3_/TiO_2_ and MoO_3_/TiO_2_ composites. Appl. Surf. Sci..

[16] Chuai H., Zhou D., Zhu X., Li Z., Huang W. (2015). Characterization of V_2_O_5_/MoO_3_ composite photocatalysts prepared via electrospinning and their photodegradation activity for dimethyl phthalate. Chin. J. Catal..

[17] Danyliuk N., Tatarchuk T., Kannan K., Shyichuk A. (2021). Optimization of TiO_2_-P25 photocatalyst dose and H_2_O_2_ concentration for advanced photo-oxidation using smartphone-based colorimetry. Water Sci. Technol..

[18] Doong R.A., Chang P.Y., Huang C.H. (2009). Microstructural and photocatalytic properties of sol–gel-derived vanadium-doped mesoporous titanium dioxide nanoparticles. J. Non-Cryst. Solids.

[19] Wang Y., Su Y.R., Qiao L., Liu L.X., Su Q., Zhu C.Q., Liu X.Q. (2011). Synthesis of one-dimensional TiO_2_/V_2_O_5_ branched heterostructures and their visible light photocatalytic activity towards Rhodamine B. Nanotechnology.

[20] Abla F., Elsayed Y., Abu Farha N., Obaideen K., Mohamed A.A., Lee H., Kanan S. (2023). Fabrication of high surface area TiO_2_-MoO_3_ nanocomposite as a photocatalyst for organic pollutants removal from water bodies. Catalysts.

[21] Alnaggar G., Hezam A., Bajiri M.A., Drmosh Q.A., Ananda S. (2022). Sulfate radicals induced from peroxymonosulfate on electrochemically synthesized TiO_2_–MoO_3_ heterostructure with Ti–O–Mo bond charge transfer pathway for potential organic pollutant removal under solar light irradiation. Chemosphere.

[22] Singh N., Jana S., Singh G.P., Dey R.K. (2020). Graphene-supported TiO_2_: study of promotion of charge carrier in photocatalytic water splitting and methylene blue dye degradation. Adv. Compos. Hybrid Mater..

[23] Etacheri V., Di Valentin C., Schneider J., Bahnemann D., Pillai S.C. (2015). Visible-light activation of TiO_2_ photocatalysts: advances in theory and experiments. J. Photochem. Photobiol. C Photochem. Rev..

[24] Khalilzadeh A., Fatemi S. (2016). Spouted bed reactor for VOC removal by modified nano-TiO_2_ photocatalytic particles. Chem. Eng. Res. Des..

[25] Maheswari N., Muralidharan G. (2017). Controlled synthesis of nanostructured molybdenum oxide electrodes for high performance supercapacitor devices. Appl. Surf. Sci..

[26] Grover I.S., Singh S., Pal B. (2013). The preparation, surface structure, zeta potential, surface charge density and photocatalytic activity of TiO_2_ nanostructures of different shapes. Appl. Surf. Sci..

[27] Kumar A., Pandey G. (2017). A review on the factors affecting the photocatalytic degradation of hazardous materials. Mater. Sci. Eng. Int. J.

